# Preliminary investigation of bacterial surface contamination in emergency ambulances in South Korea

**DOI:** 10.1186/s12245-025-01083-z

**Published:** 2025-12-05

**Authors:** Seoul-Hee Nam, Hyeon-Ji Lee, Mi-young Choi

**Affiliations:** 1 Department of Dental Hygiene, College of Health Sciences, Kangwon National University, 346 Hwangjo-Gil, Samcheok-City Gangwon-do, 25949 Republic of Korea; 2 Department of Paramedicine, College of Health Sciences, Kangwon National University, 346 Hwangjo-Gil, Samcheok-City Gangwon-do, 25949 Republic of Korea

**Keywords:** Ambulance contamination, Infection prevention, Emergency medical services, Bacterial persistence, Disinfection efficacy, Bacillus velezensis, Williamsia muralis, EMS infection control

## Abstract

**Background:**

Emergency ambulances are vital in prehospital care but carry a high risk of healthcare-associated infections due to confined spaces, high patient turnover, and brief cleaning intervals. Routine disinfection protocols are in place; however, their effectiveness in South Korean ambulances has not been formally evaluated.

**Methods:**

This pre–post observational study examined bacterial contamination on six high-touch surfaces across five operational ambulances in Province G, South Korea. Swabs were collected immediately before and after daily disinfection performed by paramedics. Bacterial load was quantified using colony-forming units (CFUs), and species identification was conducted via 16 S rRNA sequencing. Statistical analyses included paired t-tests, ANOVA, Cohen’s d, and MANOVA to evaluate the cleaning efficacy and contamination patterns.

**Results:**

All six surfaces were contaminated before cleaning, with the highest CFUs recorded on the ventilation outlet (182.6 ± 48.3), SpO₂ sensor (145.2 ± 35.7), and stretcher handle (122.4 ± 22.6). Disinfection significantly reduced bacterial load across all surfaces (*p* < 0.05), yet residual contamination remained on the SpO₂ sensor (Bacillus velezensis) and stretcher handle (Williamsia muralis). ANOVA revealed significant differences in baseline contamination (F(5,24) = 78.52, *p* < 0.001), and MANOVA confirmed that cleaning effectiveness varied by surface geometry (Wilks’ Λ = 0.202, *p* < 0.001).

**Conclusions:**

Manual disinfection significantly lowers bacterial load in ambulances, but residual contamination on complex, high-touch surfaces remains problematic. These findings underscore the need for multimodal disinfection approaches, improved equipment design, and systematic microbial surveillance to enhance EMS infection control standards.

## Introduction

Emergency ambulances are a critical component of prehospital care, providing rapid transport and early interventions for critically ill or injured patients. However, their confined space, high patient turnover, and frequent exposure to biological materials pose unique infection prevention and control (IPC) challenges. Inadequate decontamination of high-touch surfaces has been associated with healthcare-associated infections (HAIs), threatening both patient safety and the occupational health of emergency medical service (EMS) providers [1–4].

International studies have reported the presence of clinically significant microorganisms—including methicillin-resistant Staphylococcus aureus(MRSA), vancomycin-resistant Enterococcus(VRE), and Bacillusspp.—on ambulance equipment and interior surfaces [2, 5–7]. High-touch items such as stretcher handles, monitoring devices, and control panels are particularly vulnerable to contamination and may act as reservoirs for cross-transmission [8]. Importantly, residual contamination has been documented even after disinfection, often due to limited disinfectant contact time, inconsistent cleaning practices, and the structural complexity of medical equipment [8, 9].

High-income countries have responded with standardized protocols, including validated disinfectants, defined cleaning frequencies, and mandatory staff training [10–12]. By contrast, South Korea lacks nationally mandated ambulance disinfection guidelines. Current practice relies on manual cleaning performed by paramedics during short turnaround periods, supplemented by outsourced deep cleaning only once per month [13, 14]. Daily cleaning windows are often limited to 30 min, raising concerns about their adequacy.

Despite global recognition of this issue, few systematic investigations have been conducted in South Korea. Limited data exist on bacterial load in ambulances, the effectiveness of routine disinfection, and the specific species that persist after cleaning. Addressing this gap is critical in the context of rising antimicrobial resistance, increasing EMS demand, and the need to protect both patients and EMS providers.

Therefore, this study aimed to: (1) quantify bacterial contamination on high-touch ambulance surfaces, (2) evaluate the effectiveness of routine disinfection performed by EMS personnel, and (3) identify bacterial species that persist despite cleaning.

## Materials and methods

### Study design and setting

We conducted a pre–post observational study to evaluate bacterial surface contamination and the effectiveness of routine ambulance disinfection. Five ambulances were purposively selected from high-dispatch fire stations in Province G, South Korea, representing prehospital environments with high exposure risk. As the study did not involve human subjects or identifiable data, it was exempt from institutional review board (IRB) review under national research ethics guidelines. Written consent for vehicle sampling was obtained from supervising paramedics, and administrative approval was provided by each participating fire station.

### Sampling procedure

Surface swab samples were collected at two standardized time points: immediately before and immediately after routine daily disinfection performed during paramedic shift changes. On-duty paramedics were aware that environmental sampling would occur on the day of data collection; however, pre-disinfection samples were obtained prior to any additional cleaning, following only their usual routine practices. After swab collection, paramedics completed their standard post-shift disinfection, including re-cleaning of the sampled areas. All sampling was performed by a single trained investigator to minimize inter-observer variability. Sterile cotton swabs were applied over a defined 1.5 × 1.5 cm area using a circular motion.Routine cleaning was performed according to local EMS protocols. Because commercial disinfectant brands differ among fire stations, disinfectants are reported according to active ingredients rather than product names. Commonly used agents included cresol-based disinfectants and 70% alcohol-based wipes. These methods reflect current IPC practices in South Korea, where standardized national guidelines are absent.

### Sampling sites

Six high-touch surfaces were selected based on frequency of contact and potential for microbial transmission:

· Pulse oximeter (SpO₂) sensor

· Main stretcher handle

· Interior door handle

· Oxygen cylinder valve

· Crew seat

· Driver’s steering wheel

Post-disinfection samples were collected from the same sites using identical methods

### Microbiological analysis

Swabs were cultured on Luria–Bertani agar under aerobic conditions. Colony-forming units (CFUs) were counted to quantify bacterial load. Species-level identification was initially performed using standard biochemical assays and subsequently confirmed by 16 S rRNA gene sequencing at a certified clinical microbiology laboratory. Viral and fungal pathogens were not assessed.

### Statistical analysis

All analyses were conducted using SPSS Statistics version 25.0 (IBM Corp., USA). Data normality was tested with the Shapiro–Wilk method. Paired t-tests compared pre- and post-disinfection CFU counts for each surface. One-way analysis of variance (ANOVA) with Tukey’s post hoc test examined baseline differences in bacterial load among surfaces. Effect sizes were calculated using Cohen’s d. A multivariate analysis of variance (MANOVA) was used to evaluate the interaction between surface type and disinfection status. A p-value < 0.05 was considered statistically significant.

## Result

### Bacterial contamination on ambulance surfaces

Bacterial growth was detected on all six high-touch surfaces prior to disinfection, with members of the Bacillaceaefamily most frequently isolated (Table 1). The SpO₂ sensor and main stretcher handle showed the greatest species diversity, including multiple Bacillusspecies and opportunistic organisms such as Roseomonas mucosaand Moraxella osloensis.

Following disinfection, most surfaces demonstrated complete elimination of detectable bacteria. However, persistent contamination remained on two critical sites: Bacillus velezensison the SpO₂ sensor and Williamsia muralison the stretcher handle. Both surfaces are characterized by irregular geometries and frequent handling, which may reduce cleaning effectiveness.

**Table 1:** Bacterial species identified on six high-touch ambulance surfaces before and after routine disinfection (confirmed by 16 S rRNA sequencing)

Surface	Pre-Disinfection Species	Post-Disinfection Species
SpO₂ sensor	Bacillus amyloliquefaciens, B. megaterium, B. velezensis, Roseomonas mucosa, Streptomyces coelicolor, S. griseolus	Bacillus velezensis
Main stretcher handle	B. cereus, B. megaterium, Dermacoccus nishinomiyaensis, Moraxella osloensis, Terribacillus saccharophilus	Williamsia muralis
Interior door handle	B. megaterium, B. velezensis, Neobacillus niacini	None detected
Crew seat	Staphylococcus hominis, Lysinibacillus fusiformis, D. nishinomiyaensis	None detected
Ventilation outlet	B. cereus	None detected
Steering wheel	D. nishinomiyaensis	None detected

### Quantitative comparison of bacterial load before and after disinfection

**Fig. 1:**
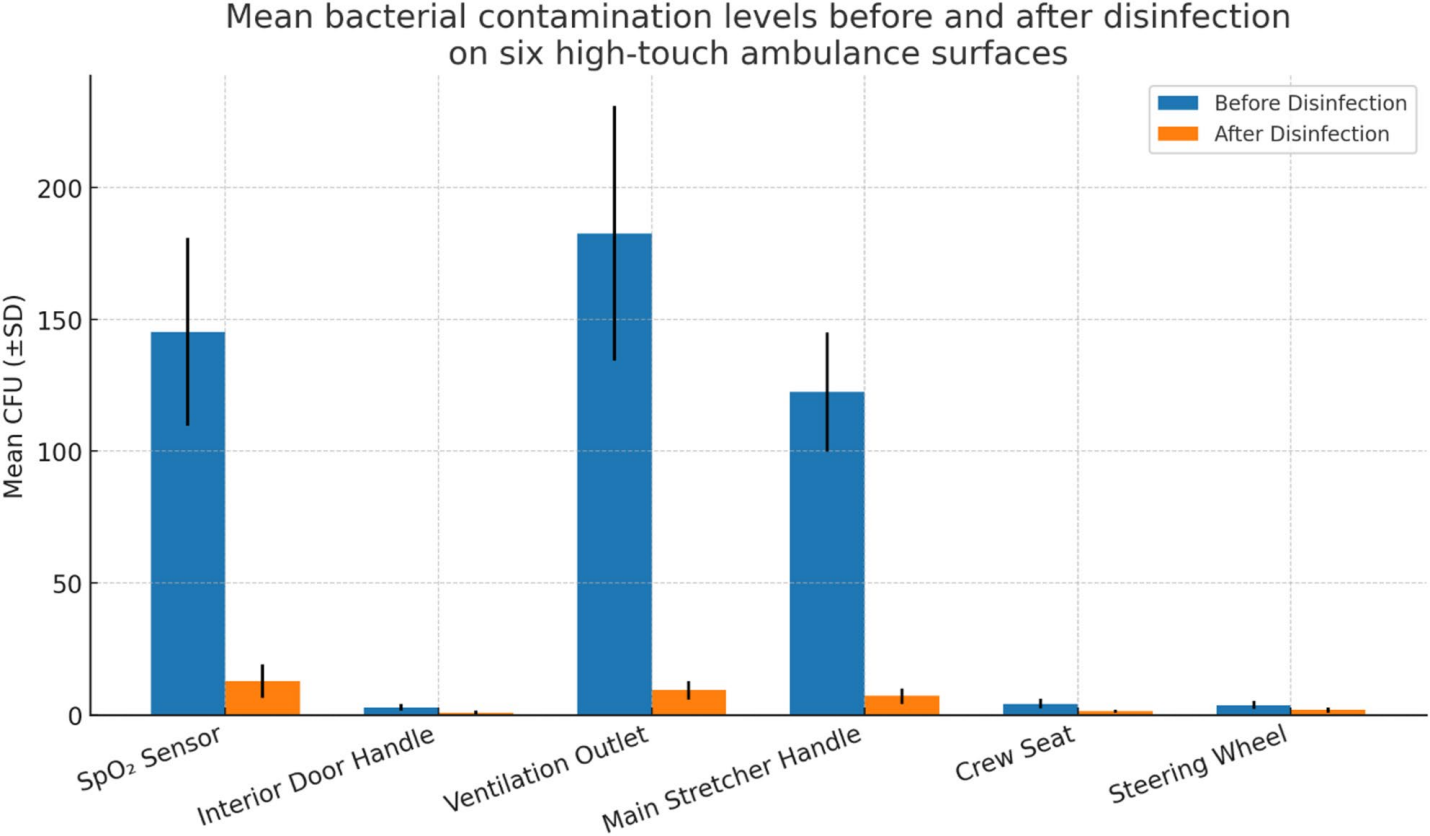
Mean bacterial contamination levels before and after routine disinfection on six high-touch ambulance surfaces. (Error bars indicate standard deviations. Significant reductions were observed across most surfaces, although residual contamination persisted on the SpO₂ sensor and stretcher handle.)

Mean CFU counts before and after disinfection are summarized in Table 2; Fig. 1. All surfaces showed bacterial reduction, though the magnitude varied by surface type.

The ventilation outlet exhibited the highest baseline contamination (182.6 ± 48.3 CFU), followed by the SpO₂ sensor (145.2 ± 35.7 CFU) and stretcher handle (122.4 ± 22.6 CFU). All showed significant reductions after disinfection (*p* < 0.001), although residual growth persisted on the SpO₂ sensor and stretcher handle. In contrast, the interior door handle (2.8 ± 1.2 CFU) and crew seat (4.2 ± 1.8 CFU) demonstrated low baseline loads and near-complete clearance (*p* = 0.041 and *p* = 0.012). The steering wheel decreased from 3.6 ± 1.5 to 1.8 ± 1.0 CFU, but this was not statistically significant (*p* = 0.057), suggesting possible recontamination or insufficient disinfectant contact time.

**Table 2:** Mean bacterial contamination levels (CFU) before and after routine disinfection on six ambulance surfaces

Surface	Time Point	CFU (Mean ± SD)	*p*-value
SpO₂ Sensor	Before	145.2 ± 35.7	< 0.001
	After	12.8 ± 6.4	
Interior Door Handle	Before	2.8 ± 1.2	0.041
	After	0.9 ± 0.7	
Ventilation Outlet	Before	182.6 ± 48.3	< 0.001
	After	9.3 ± 3.5	
Main Stretcher Handle	Before	122.4 ± 22.6	< 0.001
	After	7.1 ± 2.9	
Crew Seat	Before	4.2 ± 1.8	0.012
	After	1.3 ± 0.5	
Steering Wheel	Before	3.6 ± 1.5	0.057
	After	1.8 ± 1.0	

*Paired t-test; *p* < 0.05

### Supplementary statistical analyses

One-way ANOVA showed significant differences in baseline CFU levels among surface types (F(5,24) = 78.52, *p* < 0.001). Post hoc testing confirmed that the SpO₂ sensor and ventilation outlet carried significantly higher contamination than the interior door handle and crew seat (*p* < 0.001).

Effect size calculations (Cohen’s d) indicated very large effects for the SpO₂ sensor (3.46), ventilation outlet (3.26), and stretcher handle (4.04), and large effects for the interior door handle (1.87), crew seat (1.99), and steering wheel (1.30) (Table 3). These results support the general effectiveness of routine disinfection, while emphasizing that complex, high-touch equipment remains more difficult to decontaminate.

MANOVA confirmed a significant interaction between surface type and disinfection status (Wilks’ Λ = 0.202, F(6,19) = 12.84, *p* < 0.001), underscoring that cleaning efficacy is strongly influenced by equipment design and accessibility.

**Table 3:** Effect sizes (Cohen’s d) for CFU reduction by surface type (Effect size interpretation: >0.8 = large, > 2.0 = very large)

Surface	Cohen’s d	Interpretation
SpO₂ Sensor	3.46	Very large effect
Ventilation Outlet	3.26	Very large effect
Main Stretcher Handle	4.04	Very large effect
Interior Door Handle	1.87	Large effect
Crew Seat	1.99	Large effect
Steering Wheel	1.30	Large effect

## Discussion

This study provides one of the first systematic assessments of bacterial contamination in South Korean emergency ambulances, highlighting both the efficacy and limitations of routine disinfection practices. Although significant reductions in CFU counts were observed across all tested surfaces, residual contamination persisted on complex, high-touch equipment such as the SpO₂ sensor and stretcher handle, underscoring vulnerabilities in current infection prevention measures [6, 9].

### Effectiveness and limitations of current cleaning protocols

Manual disinfection proved effective on smooth, non-porous surfaces, with large effect sizes noted for the interior door handle and crew seat. However, multivariate analysis confirmed that cleaning efficacy varied significantly according to surface geometry and accessibility. These findings are consistent with international evidence showing that ambulances often remain contaminated despite routine cleaning [1–3, 5, 7]. Reports from prehospital and EMS studies further emphasize that cleaning windows are typically short and performed under operational pressure, limiting overall cleaning performance [8, 9, 17].

### Clinical implications of persistent organisms

The recovery of Bacillus velezensisfrom the SpO₂ sensor and Williamsia muralisfrom the stretcher handle following disinfection is clinically relevant. While these organisms are primarily environmental, they possess biofilm-forming capacity and sanitizer resistance, and have been detected in healthcare environments [15–17, 19]. The SpO₂ sensor directly contacts patient skin, including immunocompromised individuals, and stretcher handles are frequently touched by both providers and patients, creating a potential pathway for cross-contamination. These findings align with previous reports linking ambulance environments to reservoirs of opportunistic and resistant microorganisms [18].

### Implications for EMS infection control policy

The results support CDC and WHO recommendations that prehospital IPC protocols must be context-specific and risk-stratified [10, 21]. A uniform wipe-based strategy is insufficient, and a multimodal approach should be considered. For example, automated adjunct technologies such as pulsed xenon UV, ozone fogging, or hydrogen peroxide vapor have demonstrated added value in reducing microbial burden in EMS vehicles [20]. Likewise, redesigning ambulance equipment to minimize seams and recesses may decrease areas where bacteria can persist despite surface cleaning [22]. The application of antimicrobial coatings on high-touch surfaces has also been proposed as a preventive measure, particularly for equipment that is frequently handled during patient care. Finally, establishing routine microbial surveillance with structured feedback to EMS providers is critical for maintaining quality assurance and ensuring adherence to infection control standards [13]. Recent evidence further emphasizes that staff training and compliance monitoring are essential to sustain decontamination quality [11, 12]. Incorporating these measures into national EMS-specific IPC guidelines would strengthen both patient and provider safety and align practice in South Korea with international best standards.

### Future research directions

Culture-based methods, while informative, underestimate microbial diversity. Future work should employ high-throughput metagenomic sequencingto capture viable but non-culturable organisms and resistance genes [17]. Randomized controlled trials comparing manual cleaning with advanced or hybrid technologies are warranted to evaluate microbial efficacy, cost-effectiveness, and operational feasibility [13, 20]. Longitudinal studies assessing the impact of paramedic training, equipment redesign, and novel disinfectants will be essential to optimize ambulance IPC in real-world settings [7, 8, 22].

## Conclusion

This preliminary investigation shows that routine manual disinfection in South Korean emergency ambulances significantly reduces bacterial contamination but does not fully eliminate organisms on complex, high-touch equipment such as SpO₂ sensors and stretcher handles. These findings highlight a critical gap in current prehospital IPC practices. To protect both patients and EMS providers, national guidelines should adopt multimodal cleaning strategies, equipment redesign, and systematic microbial surveillance. Future research employing molecular diagnostics and randomized controlled trials will be essential to inform robust, evidence-based infection control standards for EMS.

## Data Availability

The datasets generated and/or analyzed during the current study are not publicly available because they contain no human subject data but are available from the corresponding author on reasonable request.
